# Novel Model for Comprehensive Assessment of Robust Prognostic Gene Signature in Ovarian Cancer Across Different Independent Datasets

**DOI:** 10.3389/fgene.2019.00931

**Published:** 2019-10-11

**Authors:** Zhitong Bing, Yuxiang Yao, Jie Xiong, Jinhui Tian, Xiangqian Guo, Xiuxia Li, Jingyun Zhang, Xiue Shi, Yanying Zhang, Kehu Yang

**Affiliations:** ^1^Evidence Based Medicine Center, School of Basic Medical Science of Lanzhou University, Lanzhou, China; ^2^Key Laboratory of Evidence Based Medicine and Knowledge Translation of Gansu Province, Lanzhou, China; ^3^Department of Computational Physics, Institute of Modern Physics, Chinese Academy of Sciences, Lanzhou, China; ^4^School of Physical Science and Technology, Lanzhou University, Lanzhou, China; ^5^Department of Applied Mathematics, Changsha University, Changsha, China; ^6^Medical Bioinformatics Institute, School of Basic Medicine, Henan University, Henan, China; ^7^School of Public Health, Lanzhou University, Lanzhou, China; ^8^Institute for Evidence Based Rehabilitation Medicine of Gansu Province, Lanzhou, China; ^9^Department of Pharmacology and Toxicology of Traditional Chinese Medicine, Gansu University of Chinese Medicine, Lanzhou, China

**Keywords:** ovarian cancer, prognosis index, Cox regression, gene signature, robust prognostic model

## Abstract

Different analytical methods or models can often find completely different prognostic biomarkers for the same cancer. In the study of prognostic molecular biomarkers of ovarian cancer (OvCa), different studies have reported a variety of prognostic gene signatures. In the current study, based on geometric concepts, the linearity-clustering phase diagram with integrated P-value (LCP) method was used to comprehensively consider three indicators that are commonly employed to estimate the quality of a prognostic gene signature model. The three indicators, namely, concordance index, area under the curve, and level of the hazard ratio were determined *via* calculation of the prognostic index of various gene signatures from different datasets. As evaluation objects, we selected 13 gene signature models (Cox regression model) and 16 OvCa genomic datasets (including gene expression information and follow-up data) from published studies. The results of LCP showed that three models were universal and better than other models. In addition, combining the three models into one model showed the best performance in all datasets by LCP calculation. The combination gene signature model provides a more reliable model and could be validated in various datasets of OvCa. Thus, our method and findings can provide more accurate prognostic biomarkers and effective reference for the precise clinical treatment of OvCa.

## Introduction

Ovarian cancer (OvCa) is the most prevalent lethal gynecological malignancies and the fifth-leading cause of cancer death among the female population in the United States, with 21,880 new cases and 13,850 deaths in 2009 (Jemal et al., 2010). In 2018, 22,530 new cases and 13,980 deaths were reported in USA (Siegel et al., 2019). Based on the above statistical data and the results from publications on research on OvCa, we found that during nearly 10 years, the incidence and mortality of OvCa have not significantly decreased. The main reason for this is generally considered to be the clinically heterogeneous nature of OvCa. Patients with morphologically similar, advanced-stage tumor display a broad range of clinical outcomes. Prognostic factors, including age, performance status, tumor node metastasis (TNM) stage, histological grade and subtype, and initial surgery results, are insufficient to reflect the important individual variation in response to chemotherapy and to assess survival time among various therapeutic strategies.

The aim of this study is to find a method to evaluate different prognostic gene signature in the OvCa. We found that different studies have found completely different biomarkers for the same cancer. In this era of personalized medicine, molecular biomarkers as important factors for promoting prognosis are being studied comprehensively. With increasing number of clinical cases and the availability of corresponding gene expression profiles, many studies from 2005 to 2017 have provided gene signatures for predicting prognosis of OvCa (Bonome et al., 2005; Bonome et al., 2008; Crijns et al., 2009; Denkert et al., 2009; Mok et al., 2009; Hernandez et al., 2010; Konstantinopoulos et al., 2010; Yoshihara et al., 2010; Network TCGA, 2011; Sabatier et al., 2011; Bentink et al., 2012; Gyorffy et al., 2012; Kang et al., 2012; Kernagis et al., 2012; Yoshihara et al., 2012; Verhaak et al., 2013; Riester et al., 2014; Waldron et al., 2014; Willis et al., 2016; Yang et al., 2016; Nguyen et al., 2017). Despite this, the prognosis for OvCa has not improved significantly as expected. In the above studies, various types of biomarkers were obtained by diverse statistical methods, platforms, and patient sample sets, leading to irreproducible results. For multi-platform data, meta-analysis is a very effective way to integrate data from different sources (Li et al., 2014; Ge et al., 2018). Hence, some studies extracted novel gene signature by merging datasets and meta-analysis (Riester et al., 2014; Waldron et al., 2014). Additionally, some studies applied other methods to estimate the quality of gene signature in OvCa. For instance, Waldron et al. (2014) estimated the performance of each gene signature model by comparing with the random gene group, whereas Riester et al. (2014) merged different data and provided a novel gene signature model. With the development of powerful statistical methods and the increasing amount of data, new biomarkers for predicting OvCa outcome have been put forward (Willis et al., 2016; Yang et al., 2016; Nguyen et al., 2017).

From the perspective of medical statistics, larger sample size indicates more accuracy and confidence level. Moreover, numerous clinical factors and heterogeneity of population and diseases lead to poor performance of distinction even in the same sample sizes. Even though diverse microarray platforms have made enormous progress in accuracy, variations among platforms cannot be ignored when drawing conclusions associated with key gene expression or gene signatures (Barnes et al., 2005; Crijns et al., 2009; Thomson and Dinger, 2016). Above-mentioned publications of OvCa gene signature also utilized different training or testing sample sizes and platforms, and few researchers focused on both sample practicability and risk gene universality.

Generally, the indicators for assaying the performance of prognostic model are concordance index (C-index), hazard ratio (HR), area under the curve (AUC) of receiver operating characteristic (ROC) curve, and P-value of the log-rank test. Although any one of the indicators can estimate the performance of prognostic model, it is not clear how to integrate these indicators for reasonable evaluation of the prognostic model, because each indicator represents different attributes in one model. There is insufficient evidence to show that a single index can effectively prove the stability and universality of a model. Thus, to construct a reasonable integrative evaluation method that we proposed, LCP method is needed. Moreover, when all the indicators are not uniform, it is difficult to judge whether a model is good or bad. For example, it is difficult to estimate the performance of a model with high HR and low AUC level. In the research of systematic evaluation, researchers have developed many tools to evaluate medical guidelines (Norris et al., 2016). However, it still remains difficult to estimate quality of genome research and select an appropriate gene signature model. Thus, we proposed a method to integrate these indicators and directly show the model with better performance. Firstly, prognostic index (PI) as a discrimination index for classifying patients into high-risk and low-risk groups was calculated by linear combination of gene signature expression and their coefficients from Cox regression. Secondly, HR, C-index, AUC, and P-value for PI were computed across different datasets. Thirdly, we mapped the value of HR, C-index, and AUC into three-dimensional space coordinate system and evaluated the models from geometric property, linearity, and clustering. Additionally, P-value used color depth for indicting distinguishing ability of model.

## Materials and methods

### Data Collection and Filtering Process

In order to ensure coincident statistical protocols, we only focused on models obtained from Cox regression methods. Based on this, we filtered out 13 models with gene signature by Cox regression. For assessing each model, we found 16 OvCa sample sets from two databases, The Cancer Genomic Atlas (TCGA) and Gene Expression Omnibus (GEO). The TCGA-OvCa dataset, employed by three platforms (Affymetrix HT U133a, Illumina HiSeq V2, AgilentG450), were considered as three different sub-datasets to assess the influence of platform diversity. From the GEO database, we collected 13 sample sets of OvCa patients from 2005 to 2017, namely, GSE19161 (Konstantinopoulos et al., 2011), GSE3149 (Bild et al., 2006), GSE9899 (Tothill et al., 2008), GSE26712 (Bonome et al., 2008), GSE14764 (Denkert et al., 2009), GSE18520 (Mok et al., 2009), GSE17260 (Yoshihara et al., 2010), GSE26193 (Mateescu et al., 2011), GSE32062 (Yoshihara et al., 2012), GSE30009 (Gillet et al., 2012), GSE63885 (Lisowska et al., 2014), GSE13876 (Crijns et al., 2009), and GSE19829 (Thomson and Dinger, 2016). Only samples satisfying the following three conditions were viewed as valid: (1) complete mRNA expression profile and clinical information, (2) survival information on patient, and (3) the tumor was primary tumor.

### Preprocessing of mRNA Expression Profiles Associated With Survival Analysis

The three platforms in TCGA have their own characteristics (value of Affymetrix HT U133a is positive with the magnitude of 10^0^∼10^1^, AgilentG450 has both positive and negative values, and Illumina HiSeq V2 included more zero than the other two platforms). Since there was no distinct difference between the absolute value of the three platforms, each platform was considered as an independent sample set, and we retained the original expression matrix to keep platform characteristics. The 13 sample sets in GEO were collected from published literature. Each dataset has its own research topic or purpose, test time and date, and different sample sizes. Some expression profiles are provided by probes not genes, such that one gene may map multiple probes, leading to the “several-for-one” matching phenomena. The expression levels of various probes that map the same gene are approximate; therefore, we integrated multi-probes as one gene by getting their arithmetic average to prevent repeated calculations. The same processing was implemented for multiple probe biomarkers (see below for details).

### Obtaining and Processing HR Value

The 13 collected models provided their own HR or coefficient values (β) by means of Cox regression method, and the two types of factor can be transformed by the formula β = log (HR). All gene signature models were utilized by following analysis for calculating PI/NPI in all datasets. For simplicity and consistency, all HR values were transformed into β. As mentioned above, one gene can be detected by multiple probes, which have different HRs; in such cases, we integrated the probes using mean values.

### PI Evaluation of Risk Gene Groups/Signature

PI, as an estimate of one patient’s risk, is the linear combination of risk coefficient multiplied by corresponding mRNA expression in its standard form, namely, normalized prognostic index (NPI), which can reflect one patient deviation in patient sets. We used the gene signature from each model to construct PI/NPI.

(1) $$\text{PI}=\overset{}{\underset{i}{\sum }}(\beta_{i}\times X_{i})$$

(2) $$\text{NPI}=\frac{\text{PI}-\text{mean}\left(\text{PI}\right)}{\text{SD}\left(\text{PI}\right)}$$

where X*_i_* is the value of the *i*th variable with its regression coefficient β*_i_*. For PI, X*_i_* is the mRNA expression value of each risk gene in each model, and β*_i_* is the coefficient of Cox regression of the *i*th gene. After calculating each patient’s NPI in one dataset, the median NPI was used as the cutoff point to classify patients into high-risk (with NPI greater than median value) and low-risk groups.

For assessing each gene signature model performance among various datasets, we treated each sample set as an independent one. According to the NPI of each patient, we classified patients into high-/low-risk groups. Then, we analyzed each sample set’s overall survival (OS) difference between the two groups based on Kaplan–Meier survival curves and HR with 95% confidence interval calculated by univariate Cox regression analysis based on NPI. Especially, the HR obtained here was characterized as one model’s HR and not at gene level, and one gene signature model was verified in one dataset only, resulting in one HR value. P-value of log-rank test (two-sided test) was used to determine the difference between high-risk and low-risk groups. Similarly, we also calculated the AUC of the corresponding ROC curve and obtained the C-index of each model in various datasets. For all above-mentioned data, the filtering, preprocessing, and survival analysis were done using R (V.3.5.1), (Ihaka and Gentleman, 1996), with help of the *survival* and *survivalROC* package (Heagerty et al., 2000).

### Integrated Assessment of Models From Geometric Approach: LCP Methods

The main purpose of this study was to evaluate the prognostic ability of various OvCa risk models in an integrated geometric method. Therefore, using collinearity verification (**Supplementary Figure S1**), we integrated the indicators, namely, HR, C-index, and AUC as model-in-dataset coordinates. In detail, we considered one model’s performance in all 16 datasets as a group of three-dimensional (3D) scatter points in clinical indicator space (all three indexes of HR, C-index, and AUC as independent coordinates). We transformed the three factors into same interval as follows:

(3) HR’=logHR

(4) $${\text{C}}_{index}^{'}=\log\left(2\;\times \;{\text{C}}_{index}\right)$$

(5) AUC′=log(2 × AUC)

Thus, trivial values of HR, C-index, and AUC (1.0, 0.5, and 0.5 respectively) would be transferred into zero. For the 3D scatter points, we proposed two geometric concepts—linearity and clustering—reflecting one model’s consistency and robustness, respectively. The residual of the best fitting 3D straight line through all scatter points stands for linearity, and clustering was obtained by 3D scatter points’ first moment and second moment (see the following equations):

(6) $$Residual=\underset{i}{\sum }DL{\left(x,y,z\right)}_{i}$$

where DL is a function that calculates the distance between one 3D point and the best fitting line in one model, and the summation notation indicates consideration of each point (the 16 datasets) together to obtain the model’s residual; x, y, and z are HR,’ C-index,’ and AUC’ values of one model in one specified dataset, respectively; ${\vec{r}}_{i}={\left(x,y,z\right)}_{i}$ is the vector representation of 3D points, while $\vec{r}$ is all scatter points’ geometric center point (first moment, the average values of x, y, and z, respectively); the summation of second-order distance from all scatters to the center point is considered as clustering (second moment). Besides, Num is total number of datasets, and *i* means analyzing all 16 datasets in an ergodic system. According to the two new indexes of one model and overall P-values representing the mean confidence level of the model in different datasets, we drew each model in two-dimensional linearity-clustering phase diagram with confidence level color bar. This linearity-clustering phase diagram with integrated P-values, called as LCP, can assist us with an all-round analysis of one model’s performance. Moreover, it can guide in the discovery of novel and effective models.

### Gene Ontology Enrichment

The genes included in each prognostic model may imply some clear or potential mechanism. Based on this consideration, gene function enrichment was analyzed using the online tool Metascape (Tripathi et al., 2015). This tool is utilized to explore the biological process (BP) and molecular function (MF) of risk genes. Fisher’s test was used to estimate significant enrichment. Gene enrichment visualization was done using ggplot2 package of R (Wickham, 2015).

### Novel Model Construction

**Figure 1:**
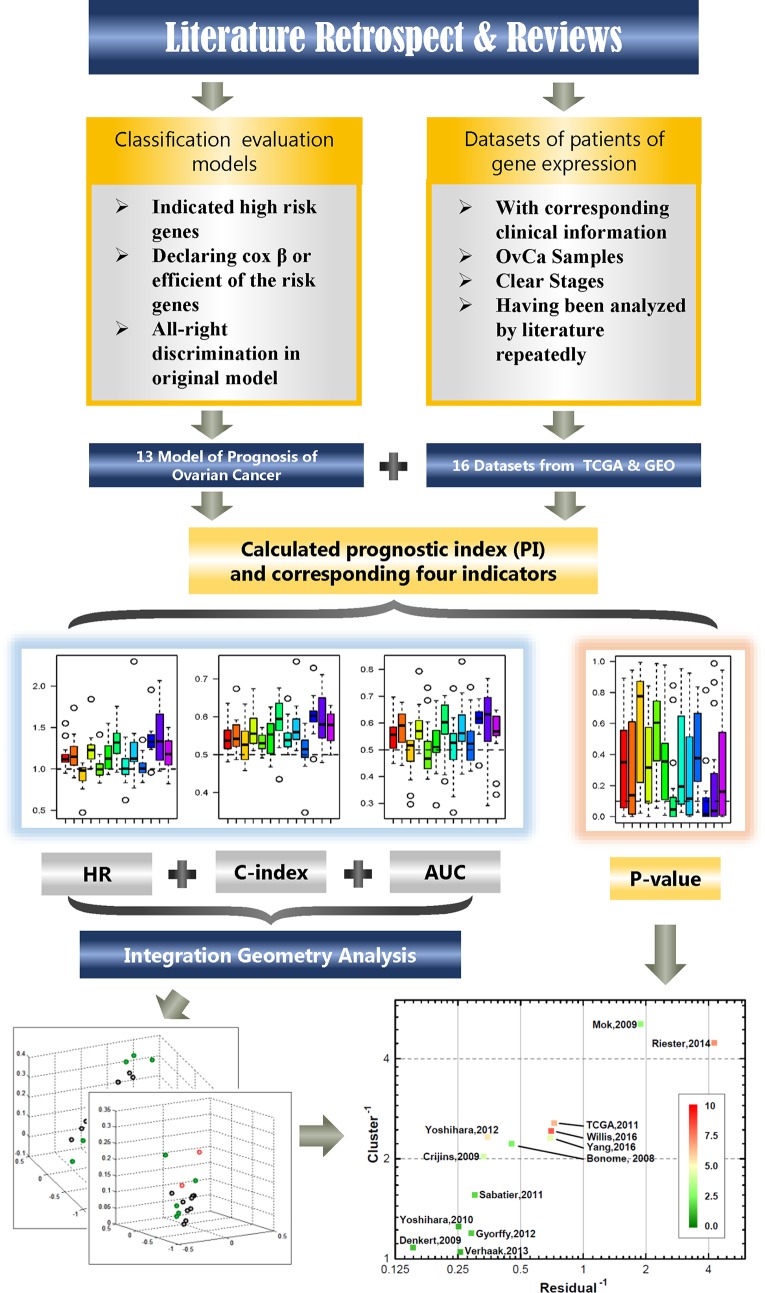
The study flowchart. The chart describes the process of filtering available dataset and method of calculation and analysis.

Using the LCP method, 13 models can be ranked. Among them, the top three models (rank three on the top of the axis) are selected and combined to get a novel model. And the novel model was validated among 16 independent datasets. For clarity, the workflow of the complete analysis process is shown in **Figure 1**.

### Statistic Analysis

In this study, P-value ≤ 0.05 was considered to be significant for log-rank test. The value of AUC and C-index >0.6 was considered as good performance. HR, and 95% confidence interval (CI) were calculated to identify low-risk (HR < 1) or high-risk gene signature model (HR > 1). Kaplan–Meier curve was employed to estimate the differences between the high- and low-risk patients.

## Results

### Characteristics of Collected Gene Signature Models and OvCa Sample Sets

**Figure 2:**
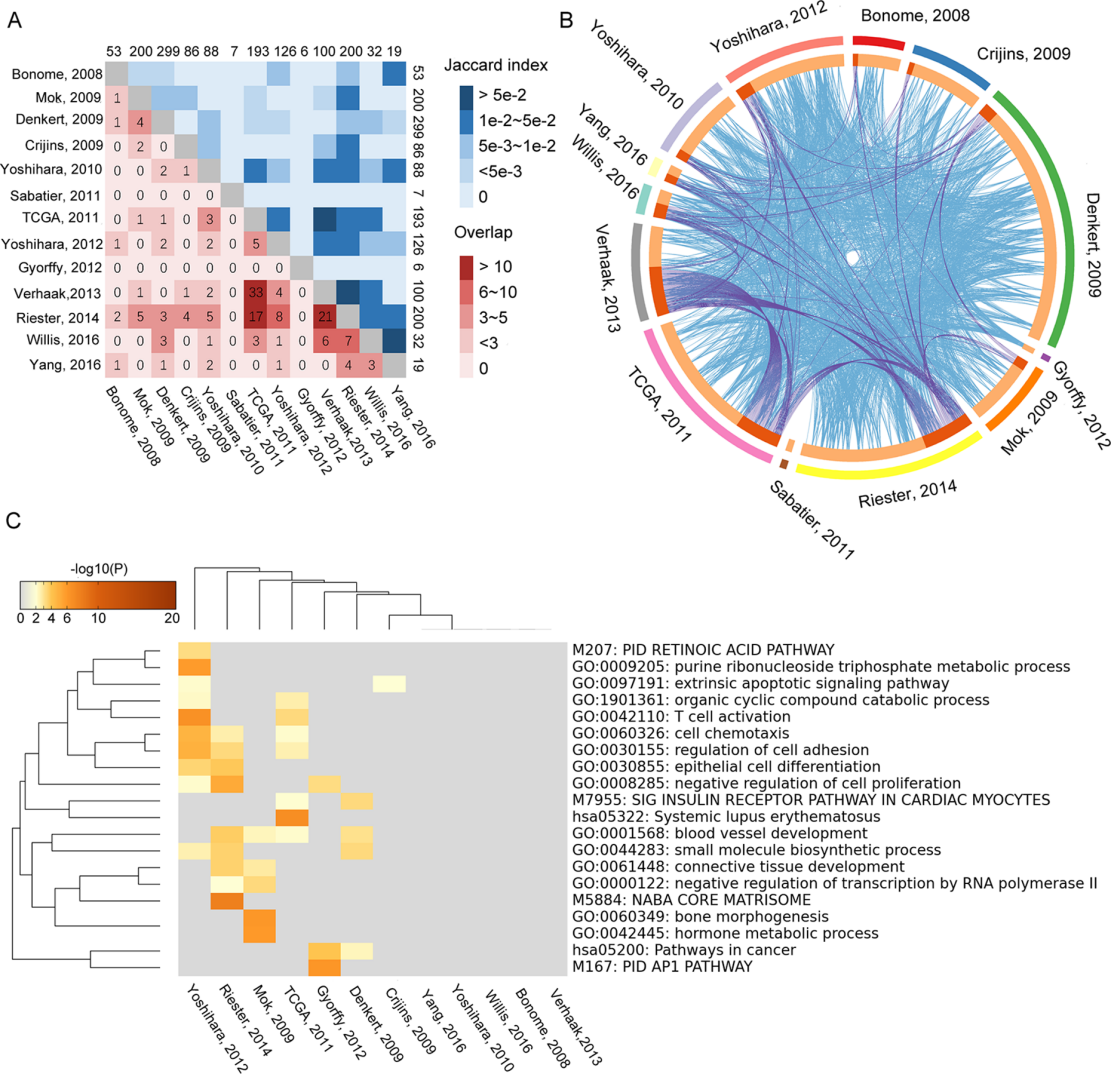
Overlapping of 13 gene signature models that were tested by Jaccard index. The numbers on edges represent the number of genes in each model. **(A)** The numbers in lattices represent the number of genes overlapping between different models. **(B)** The relationship of function among the 13 gene signature models. The blue lines represent the connection between two gene signature models. The purple lines represent at least three models associated with each other. **(C)** The overlapping of GO enrichment and KEGG among 13 gene signature models.

By searching and screening the literature, 13 prognosis gene signature models were included in this study; all of them were obtained from Cox regression method. The publications are listed in **Table 1**. Each study provided its own gene signature, with corresponding coefficients (β) or HRs. However, the 13 models showed nearly no overlap for any two models (**Supplementary Table S1**). By calculating the Jaccard indexes of pairwise models, the overlap of the vast majority of gene signature models was found to be less than 5% (**Figure 2A**). The result of GO enrichment showed that various gene signature models were enriched in different GO terms (**Figures 2B, C**).

**Table 1:** The 13 published gene signatures for the prognosis of ovarian cancer selected for meta-analysis.

Gene signature	Number of genes	Number of samples	Form of parameter	TNM stage	Grade	Dataset
Bonome et al., 2008	57	195	β	III, IV	High grade	GSE26712
Mok et al., 2009	200	53	HR	III, IV	3	GSE18520
Denkert et al., 2009	300	80	β	III, IV	2, 3	GSE14764
Crijns et al., 2009	86	110	HR	III, IV	1, 2, 3	GSE13876
Yoshihara et al., 2010	88	157	β	III, IV	1, 2, 3	GSE17260, GSE9891
Sabatier et al., 2011	7	35	HR	I, III, IV	1, 2, 3, NI	–
Network TCGA, 2011	193	489	β	II, III, IV	1, 2, 3	TCGA,2011
Yoshihara et al., 2012	126	300	β	III, IV	2, 3	GSE32062
Gyorffy et al., 2012	37	1287	HR	I, II, III, IV	1, 2, 3	TCGA, GSE14764, GSE15622, GSE19829, GSE3149, GSE9891, GSE18520, GSE26712
Verhaak et al., 2013	100	489	β	II, III, IV	High grade	TCGA, GSE9899
Riester et al., 2014	200	1525	β	I, II, III, IV	High grade	TCGA, E.MTAB.386, GSE12418, GSE13876, GSE17260, GSE18520, GSE19829, GSE26710, GSE30009, GSE32062, GSE9891
Willis et al., 2016	32	1757	HR	III, IV	High grade	TCGA, GSE14764, GSE15622, GSE19829, GSE3149, GSE9891, GSE18520, GSE26712
Yang et al., 2016	19	484	HR and β	I, II, III, IV	1, 2, 3	TCGA, GSE9899

The number of sample sets obtained after filtering is presented in **Table 1**. Overall, many studies focused on late-stage (III, IV) and high-grade (2, 3) OvCa. Notably, of these gene signature models, there were three publications that integrated data from early-stage (I, II, or II) and late-stage (III, IV) OvCa.

### Evaluation of Each Model Using 16 Independent Datasets

**Figure 3:**
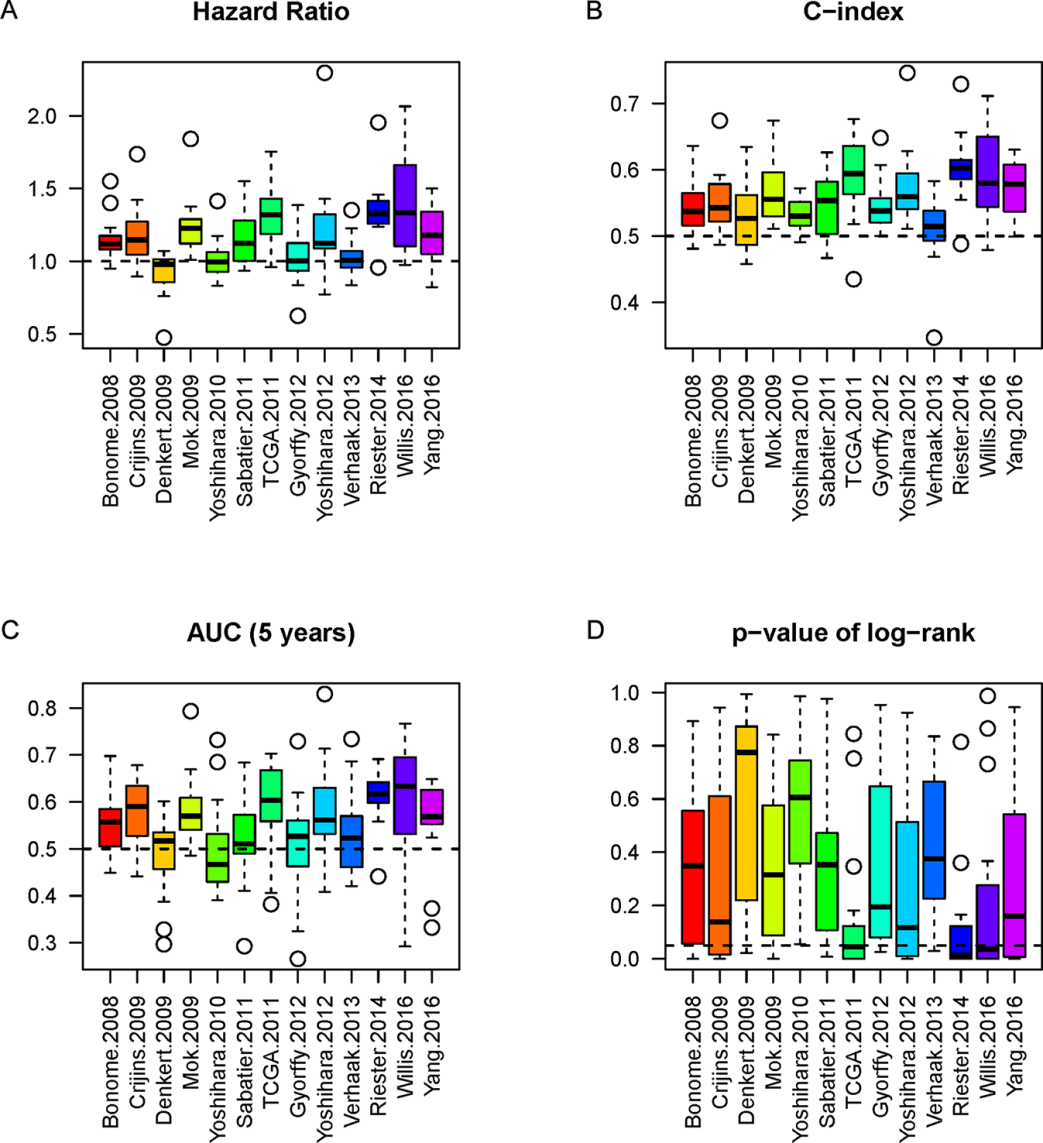
The boxplots of the three indicators and p-value. The three indicators and p-value, HR **(A)**, C-index **(B)**, AUC **(C)**, and P-value **(D)** of each model of the 16 datasets are depicted by boxplot, respectively. The dashed line represents the threshold for each indicator (HR = 1 as the threshold, C-index = 0.5 as threshold, AUC = 0.5 as threshold, and P-value = 0.05 as significant threshold). The P-values of log-rank **(D)** from comparison of high-risk and low-risk cohorts are also considered.

The NPI of each gene signature was calculated to label high or low risk for patients in each model. In order to evaluate the performance of a model sufficiently, we analyzed the model’s three indicators (HR, C-index, and AUC) simultaneously and obtained the corresponding P-value of log-rank from significance of OS between high-risk and low-risk cohorts. Boxplots were employed to show the actual distribution and distinction of the three indicators and P-value. The results showed the indicators (including AUC, C-index, p-value, and HR) represented the difference among different models (**Figure 3**). It is difficult to estimate the performance of each model by single indicator alone.

### Integrated Indicators for Re-Estimated Prognostic Gene Signature in Ovca With LCP

**Figure 4:**
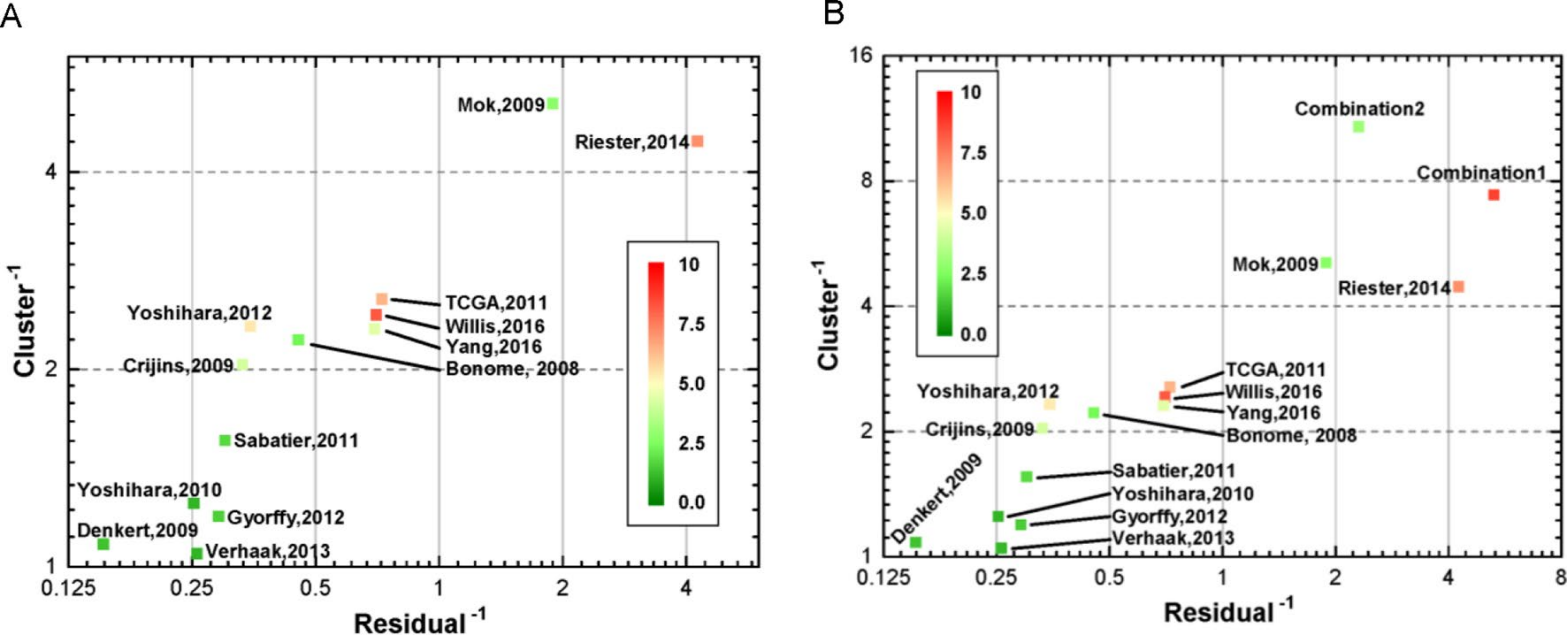
Two-dimensional linearity-clustering phase diagram of models. Tags in the figure denote the various models as described in **Table 1**. The coordinates show the reciprocals of residual of a line fitting and gathering degree of scatter points of one model in 3D space repetitively. The color of point reflects the overall confidence level. **(A)** Distribution of the 13 gene signature models in linearity-clustering phase diagram. **(B)** Distribution of the combination models in linearity-clustering phase diagram.

For investigating the relationship between the indicators, we analyzed their correlation. As presented in **Supplementary Figure S1**, HR, C-index, and AUC showed strong collinearity. In contrast, the P-value derived from log-rank test showed some negative correlation. As a consequence, the three collinearity indicators could be mapped into three-dimensional space, and we analyzed their linearity and clustering property to evaluate models from a geometric viewpoint (see Method). In this study, a novel geometric estimating method was proposed for assessment, based on which, we drew the two-dimensional linearity-clustering phase diagram of all modals analyzed (**Figure 4**). For showing an obvious distinction among the models, we obtained the reciprocals of the linearity fitting residual and clustering results, which represent the gathering degree of scatter points of one model in HR-C index-AUC (HCA) space. The model points located in top right reflected the model performing with high consistency and robustness. The color labels show the integrated P-values of one model and indicate the mean confidence level. As shown in **Figure 4A**, we found that two models (Riester and Mok) showed good clustering and linearity properties. Meanwhile, three models (Willis, Riester, and TCGA) had higher confidence level than the other models. This prompted us to examine whether combining different independent models with good performance into a novel model might improve the performance further. Therefore, two new models, called WRT (Willis+Riester+TCGA, Combination 1, **Supplementary Table S2**) and RM (Resier+Mok, combination 2, **Supplementary Table S3**), were obtained and verified (**Figure 4B**). The results showed that both WRT and RM models had good performance in the HCA space; they both appeared in the upper right position. Although RM model showed higher position than WRT, it had lower confidence level with green color. On account of this outcome, only WRT was considered as the more accurate model that can satisfy all estimation conditions. The list of genes in WRT is listed in **Supplementary Table S4**.

### Validation Results From Geometric Methods by Clustering Each Single Indicator

**Figure 5:**
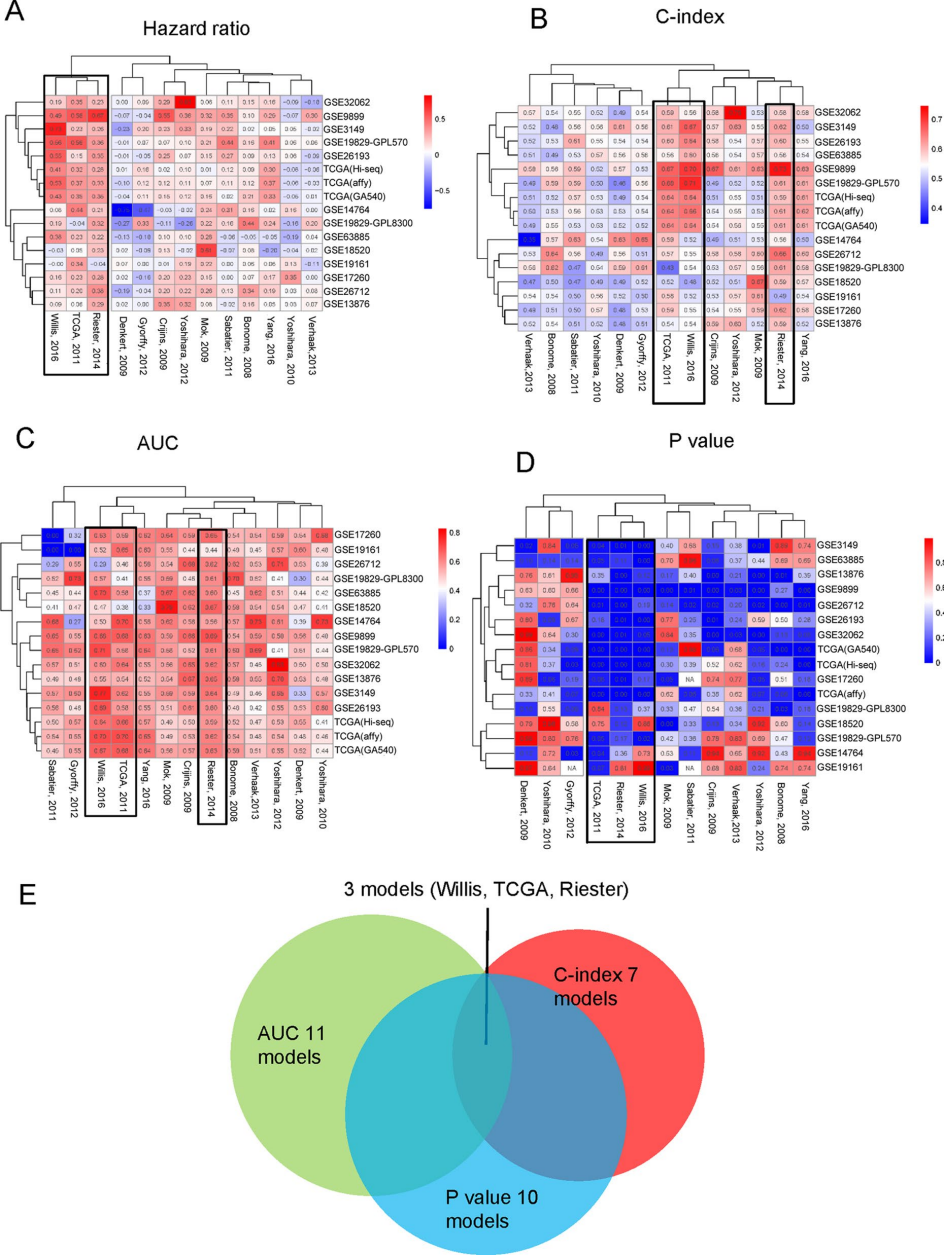
Three indicators clustered across 16 different datasets: **(A)** HR, **(B)** C-index, **(C)** AUC of 5 years. **(D)** Log-rank p-value of the 13 models clustered in 16 different datasets. **(E)** The overlapping of the four indicators.

These four indicators (HR, AUC, C-index, and p-value) were individually clustered by unsupervised hierarchical clustering in R software using the package “pheatmap” (**Figures 5A–D**). As shown in **Figure 5**, the different indicators always clustered into two groups—those that performed well or did not. Surprisingly, we always found three models, namely, Willis, TCGA, and Riester, included in the well-performing groups, irrespective of the indicators (**Figure 5E**).

### Gene Ontology Enrichment

**Figure 6:**
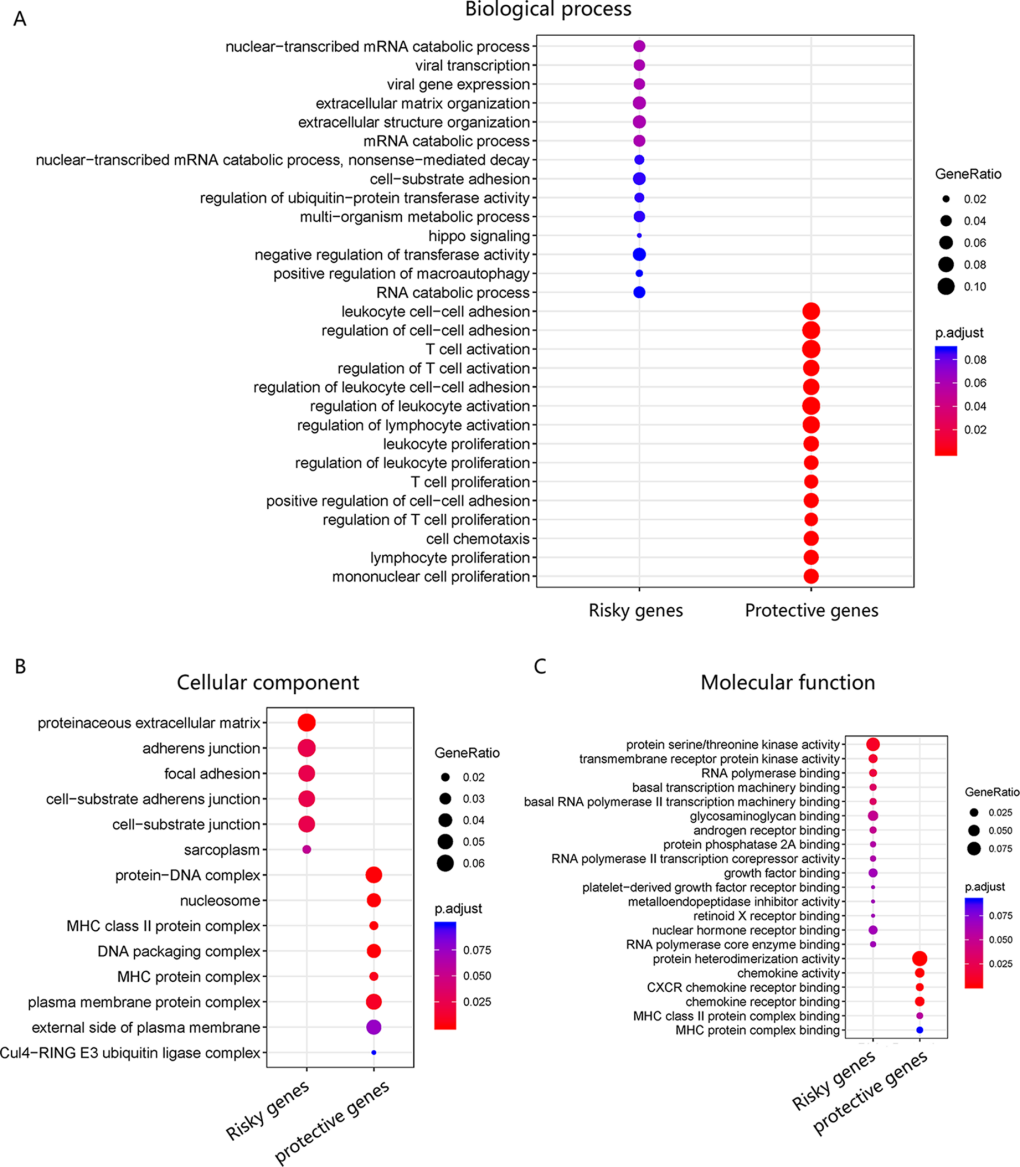
GO enrichment of WRT model. **(A)** Risky and protective gene enrichment in biological processes. **(B)** Cellular component and **(C)** molecular function.

The pathway of genes in the novel WRT combination model was analyzed for GO enrichment (**Figures 6A, B, C**), and results showed that the risky genes were mainly involved in viral transcription and viral gene expression and were associated with viral infection.

## Discussion

In this study, we evaluated 13 gene signature models from 16 datasets between 2005 and 2017. Although each prognostic model was derived from Cox regression method, some models were computed through a single dataset, while others were obtained through meta-analysis of integrated datasets. Meta-analysis has the advantage of stably merging many studies—for example, the studies by Riester et al. (2014) and Willis et al. (2016) employed meta-analysis to obtain prognostic gene signature. Although the quality of research from different regions and laboratories are very different, some studies have found that there was no significant difference in the study of genetic biomarkers between different regions (Tian et al., 2017). Importantly, some high-throughput experiments also derived good gene signatures by high-quality datasets and advanced algorithms. For example, the studies by Yoshihara et al. (2012) and Yang et al. (2016) relied on high-quality data to obtain good gene signatures. Moreover, we found that more accurate gene signature and stability results were mainly obtained from larger sample size and advanced algorithm application to some degree (**Table 1** and **Figure 5**). In 2014, Waldron et al. and Riester et al. extensively evaluated various gene signature models from previous publications for predicting outcome of patients with OvCa. In 2016, Willis et al. and Yang et al. proposed two new gene signature models for predicting prognosis of OvCa by different methods. Noticeably, there is little intersection between the two gene signatures (**Figure 2A**). Willis et al. employed meta-analysis and then verified the results in many datasets, while Yang et al. only used the TCGA dataset to train a gene signature model by elastic regulation network. From the results of clustering all gene signatures, there were five models that showed good universality. Generally, the accuracy of predictor estimation for cancer is effected by numerous practical factors, such as methodological heterogeneity, clinical heterogeneity, small sample sizes, batch effects, and lack of independent dataset for validation (Simon, 2005; Baggerly et al., 2008; Dobbin et al., 2008; Sabatier et al., 2009; Koscielny, 2010; Leek et al., 2010; Medicine, 2012). This prompted us to investigate 13 gene signature models across large gene expression datasets.

Based on geometrical concepts, our novel method, proposed in this study, integrated three indicators to estimate prognostic models. As a result, we could not only estimate each model quantitatively and give each model a position intuitively but also selected good models to combine into a new model. The results showed that the combination model (WRT model) could perform better than the individual models across the 16 independent datasets. The RM combination model, although good, did not perform as well as the WRT model.

For further explaining the role of WRT model in OvCa, we applied GO enrichment for assaying the genes in the WRT model. We found that risky genes were mainly involved in viral transcription and viral gene expression processes, while protective genes were mainly involved in immune-related processes. Both the biological function and computational results showed that the WRT model exhibited pathways associated with viral infection. Although some cancers are caused by viral infections, the relationship between OvCa and viruses is unclear. However, some researchers have reported that patients with OvCa in Indian population are infected with human papillomavirus (Shanmughapriya et al., 2012). Other researchers have found a relationship between Chlamydia and the risk of ovarian cancer (Trabert et al., 2018).

## Conclusions

In summary, our work provides a platform for further investigating the causes of different gene signatures for effective OvCa prognosis. We not only provide a method for quantitatively estimating a prognostic model and give each model an intuitive position but also propose a way to obtain a robust model for predicting prognois of OvCa. Noticeably, the integrative model from geometric approach performed better than all original models. Importantly, the multi-platform cross-database combination can obtain more realistic results.

## Data Availability Statement

Publicly available datasets were analyzed in this study. This data can be found here: http://xena.ucsc.edu/welcome-to-ucsc-xena/. TCGA: https://xenabrowser.net/datapages/. GEO: https://www.ncbi.nlm.nih.gov/geo. MetaScape: http://metascape.org/gp/index.html#/main/step1.

## Author Contributions

Conceptualization, ZB, YY and JX; Formal analysis, YY; Investigation, ZB, JZ and XS; Methodology, JT and XG; Project administration, XS; Software, YY; Supervision, KY; Validation, XL; Visualization, YY and YZ; Writing – original draft, ZB and YY; Writing – review and editing, YZ and KY. All authors read and approved the final version of the manuscript.

## Conflict of Interest

The authors declare that the research was conducted in the absence of any commercial or financial relationships that could be construed as a potential conflict of interest.

## Supplementary Materials

Figure S1 Collinearity test of three indicators among 13 gene signature models.

Table S1 The list of genes in the 13 gene signature models.

Table S2 The results of the performance of combination 1 model across 16 independent datasets (WRT).

Table S3 The results of the performance of combination 2 model across 16 independent datasets (RM).

Table S4 Integration model from three great models in OvCa.
