# Supplementary figures and images for: Novel Model for Comprehensive Assessment of Robust Prognostic Gene Signature in Ovarian Cancer Across Different Independent Datasets
Source: Front Genet. 2019 Oct 11;10:931. doi: 10.3389/fgene.2019.00931 (PMC6798149; doi:10.3389/fgene.2019.00931)

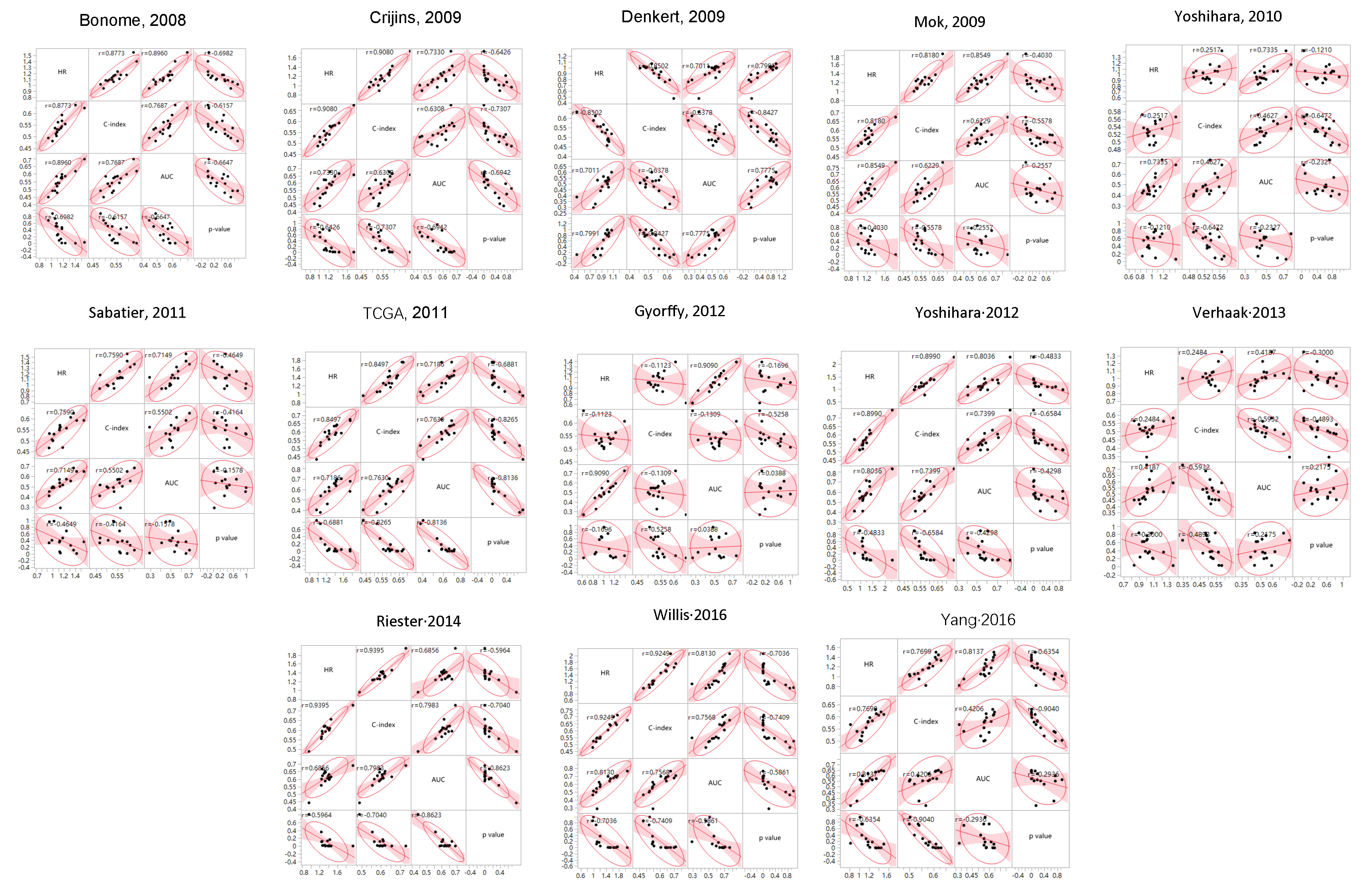

Supplement: Figure S1 — Collinearity test of three indicators among 13 gene signature models. [file Image_1.tiff]
